# Immunological detection of the Weligama coconut leaf wilt disease associated phytoplasma: Development and validation of a polyclonal antibody based indirect ELISA

**DOI:** 10.1371/journal.pone.0214983

**Published:** 2019-04-09

**Authors:** Chamini Kanatiwela-de Silva, Malini Damayanthi, Nalin de Silva, Rohana Wijesekera, Matthew Dickinson, Devaka Weerakoon, Preethi Udagama

**Affiliations:** 1 Department of Zoology and Environment Sciences, Faculty of Science, University of Colombo, Colombo03, Sri Lanka; 2 Sri Lanka Institute of Nanotechnology, Nanotechnology & Science Park, Mahenwatte, Pitipana, Homagama, Sri Lanka; 3 Department of Chemistry, Faculty of Science, University of Colombo, Colombo 03, Sri Lanka; 4 Coconut Research Institute, Lunuwila, Sri Lanka; 5 School of Biosciences, University of Nottingham, Sutton Bonington Campus, Loughborough, United Kingdom; Academia Sinica, TAIWAN

## Abstract

Weligama coconut leaf wilt disease (WCLWD) causes heavy losses in the coconut cultivations of southern Sri Lanka. The in-house developed and validated indirect ELISA was based on specific polyclonal antibodies raised in female New Zealand White rabbits, against partially purified WCLWD associated phytoplasma. This ELISA has the potential to distinguish *secA* PCR confirmed, WCLWD associated phytoplasma positive palms from phytoplasma free palms at high accuracy (93%) and sensitivity (92.7%), but with marginal specificity (79%). The calculated ELISA cross reactivity index (CRI) values were low for sugarcane white leaf (7%) and sugarcane grassy shoot (8%) infected leaves, but with marked highCRIfor both Bermuda grass white leaf (69%) and areca nut yellow leaf (70%) infected leaves. *SecA* gene based phylogenetic relationships of the WCLWD associated phytoplasma with these other locally prevalent phytoplasma strains elucidated this immunological cross reactivity, which was further reiterated by virtual restriction fragment length polymorphism analysis. Based on scanning electron microscopy, this study provides additional visual evidence, for the presence of phytoplasmas in WCLWD infected tissues.

## Introduction

Coconut (*Cocos nucifera* L.), an important tropical perennial crop, is a major source of export revenue to Sri Lanka that provides livelihood to more than 0.8 million people of the island [1]. It is a key food crop in the country responsible for about 22% of the per capita caloric intake in the diet, being second only to rice, the staple food of Sri Lankans [2]. One of the oldest agro-based industries of Sri Lanka, coconut cultivation, spans approximately 402,649 ha and accounts for 21% of agricultural lands of the island [2].

Over the last decade, the main threat to the coconut cultivation of Sri Lanka was a sudden outbreak of a rapidly spreading syndrome, first reported in 2006 from the Weligama area in the Southern Province, which was named “Weligama Coconut Leaf Wilt Disease” (WCLWD) [3,4]. WCLWD is thus far restricted to the southern province of the island. Following this initial report, a three-kilometer-wide boundary zone was declared encircling the infected area in the Southern province, and all coconut palms suspected to be infected with WCLWD within this zone were felled and removed in order to maintain a WCLWD free buffer zone. Although this precautionary measure significantly contributed towards the disease management, some spread of the disease beyond the boundary was detected in a few localities within the four-km-belt beyond the boundary [5]. Thus, the risk of spreading this disease syndrome to the “coconut triangle” in the island that encompass the western and north western provinces, which consist of more than 70% of the total land under local coconut cultivation, is not completely eliminated. The Coconut Research Institute of Sri Lanka (CRISL) was compelled to fell and destroy coconut palms in the Southern Province of the country with suspected WCLWD infection based on symptoms [5], as injecting tetracycline into the trunk of the coconut palm as a systemic treatment was neither practical nor economically viable [6,7]. Currently, the CRISL uses quantitative PCR to confirm symptomatic diagnosis of WCLWD [5].

This disease syndrome is typified by leaf flaccidity followed by intense yellowing of lower whorls of the fronds and subsequent downward bending of unopened spear leaves. With the disease progression, a reduced production of female flowers results in low productivity of fruits or nuts [4]. Previous study that reported on clear symptom remission upon tetracycline treatment, suggested that the associated pathogen was a phytoplasma [4]. Sequence analysis of the 16S ribosomal RNA (16S rRNA) gene revealed that a phytoplasma grouped under the 16SrXIand ‘*Candidatus* Phytoplasma oryzae’ related was associated with the WCLWD [4]. Conversely, subsequent multilocus sequence typing (MLST) analysis indicated that the *sec*A gene of WCLWD was related to the Bermuda Grass White Leaf Disease (BGWLD) phytoplasma of the16SrXIV group (*Candidatus* Phytoplasma cynodontis) [8].

Phytoplasmas are pleomorphic, nutritionally fastidious, plant pathogenic mollicutes associated with thousands of plant diseases worldwide. In the environment, these trans-kingdom pathogens are transmitted mainly by phloem feeding hemipteran vectors [9]. Phytoplasmas cause a wide array of economically significant diseases affecting hundreds of plant genera [10].

Sensitive and accurate detection is a prerequisite for the study, control and management of phytoplasma associated diseases. Several techniques have been used to detect phytoplasmas. Although microscopy offers a useful tool, it is less frequently used due to its limitations in differentiating phytoplasma bodies from other microorganisms or cell components such as mitochondria and chloroplasts [11]. Though DNA based molecular methods such as PCR, RFLP and DNA hybridization provides more sensitive assays [12], these are associated with inherent disadvantages of requiring specialized equipment and trained personnel.

In contrast, serologic detection of phytoplasmas using specific antiserum was used as an economical and convenient method that allows analysis of many samples within a short time period [13]. Phytoplasma specific antibodies were first produced in 1974, with the use of a partially purified phytoplasma preparation as the immunogen [13,14]. Since then, enriched or partially purified phytoplasma preparations from infected plants have been used to raise polyclonal and monoclonal antibodies against several phytoplasma strains [13,15,16].

Currently, phytoplasma strains are classified within the *‘Candidatus* Phytoplasma’ genus, principally based on at least 97.5% sequence identity within their 16S rRNA gene [17]. MLST analysis using the 16S rRNA gene and less conserved protein-encoding genes such as ribosomal protein (*rp*), *secA* and *secY* were used for the finer differentiation of distinct strains of many phytoplasma groups [18–20].

This study aimed to develop and validate an indirect ELISA based on specific polyclonal antibodies raised in rabbits to the partially purified WCLWD associated phytoplasma, to be subsequently developed as a rapid diagnostic method. Relationship of the WCLWD associated phytoplasma with other locally prevalent phytoplasma strains established through this indirect ELISA, were reiterated by their *secA* based phylogenetic relationships and by Restriction Fragment Length Polymorphism (RFLP) analysis. For the first time, Scanning Electron Microscopy provided additional visual evidence for the possible association of phytoplasma like bodies with the WCLWD.

## Materials and methods

### Plant material

Spear leaf samples of coconut palms (*Cocos nucifera* L.) manifesting symptoms of WCLWD were collected from affected coconut estates in the Weligama area (5°58′26″N, 80°25′46″E) in the Matara district (N = 207), Sri Lanka. Symptomless coconut palms (N = 192) were selected from the Rambukkana area, located adjoining the coconut triangle (6^o^15'00'' N, 80^o^ 29' 00''E) in the Kegalle district of the island.

Young sugarcane (*Saccharum officinarum* L.), leaf samples exhibiting symptoms of white leaf disease (SCWLD) (N = 30) and grassy shoot disease (SCGSD) (N = 25) were collected from fields of the Sugarcane Research Institute, Udawalawe, Sri Lanka (6°26′18″N, 80°53′18″E), while Bermuda grass (*Cynodon dactylon* L.) manifesting white leaf disease (BGWLD) (N = 25) was collected from Wadduwa (6°42′48″N,79°54′15″E) in the Kalutara district in Sri Lanka. In addition, areca palm (*Areca catechu* L.) leaf samples infected with yellow leaf disease (AYLD) (N = 35) were obtained from sites in the vicinity of coconut palms infected with WCLWD. Plant material infected with SCWLD, SCGSD, BGWLD and AYLD was used as disease controls to investigate the cross reactivity of the developed ELISA.

### PCR amplification of phytoplasma DNA

Phytoplasma positive and negative coconut spear leaf samples were collected based on WCLWD symptoms, and were confirmed for the presence of the WCLWD associated phytoplasma, using specific *secA* based PCR screening [20]. The presence of phytoplasma in other symptom positive plant material (sugarcane, Burmuda grass, areca nut palm) was also confirmed using this same PCR screening method.

Briefly, total plant DNA was extracted from samples using the small-scale DNA extraction method [21]. The quality of the extracted DNA was determined by agarose gel electrophoresis. Total DNA samples were initially analyzed by direct PCR using a phytoplasma specific universal primer pair: SecAfor1/SecArev3 [20]. A tenfold dilution of this amplicon was used as the template in the subsequent nested PCR using RicesecAfor2/ RiceseAcrev3 [22] primers specific for phytoplasma of the 16SrXI and 16SrXIV groups.

Amplification was carried out in both PCR cycles with an initial denaturation at 94 ^o^C for 2 min, 1 cycle followed by amplification for 35 cycles at 94 ^o^C for 30 seconds, 53 ^o^C for 60 seconds and 72 ^o^C for 90 seconds, and the final extension was at 72 ^o^C for 15 minutes. Both reactions were conducted in an automated thermocycler (Eppendorf Mastercycler, Germany) in a total volume of 25 μL of reaction mixture using 12.5 μL of MangoMix–PCR MasterMix (Bioline, UK) with 1 μL of DNA (100 ng/μL) and 1 μL of each primer (10 mM). Sterile distilled water served as the negative control. The nested PCR amplicons were analyzed on 1% agarose gels containing ethidium bromide. Finally, ten PCR products were purified using QIAquick PCR Purification Kit (Qiagen, USA) according to the manufacturer’s instructions and were subjected to single read sequencing at Eurofins Genomics, UK.

### Partial purification and enrichment of phytoplasma

WCLWD associated phytoplasma was purified and enriched from the spear leaves of PCR positive coconut palms [23]. Briefly, thoroughly washed non-chlorophyllous spear leaf samples (50 g) of WCLWD phytoplasma positive and negative palms confirmed by *secA*-based PCR were diced, refrigerated at 4 ^o^C for 30 min and homogenized in 150 mL of isolation medium containing 0.3 M D-mannitol, 4mM L-cysteine, 30 mM (N-morpholino) propane sulfonic acid (MOPS) buffer, 1 mM ethylene diamine tetra acetic acid and 1% (EDTA), polyvinylpyrrolidone (PVP) (pH 7.2), and ground using an electric grinder (KA-MIXEE, Singer). The filtrate of this homogenate was subjected to alternative low (1,500 *g* for 8 min) and high speed (35,000 *g* for 30 min) centrifugation (Dorval WX floor centrifuge, Thermo Fisher Scientific, UK) using a fixed angle rotor. The pellet was dissolved in 60 mL of suspending medium containing 0.3 MD-mannitol and 20 mM MOPS buffer, pH 7.0, and was subjected again to low and high-speed centrifugation. The final pellet in 5 mL of suspending medium was centrifuged at 1,500 *g* for 8 min to remove any undissolved particles. Spear leaf samples collected from symptomless coconutpalms and plants infected with other phytoplasma were also treated alike.

Density medium containing nine parts of Percoll and one part of 2.5 M sucrose (v/v), and diluting medium containing 0.25 M sucrose and 10 mM MOPS buffer, pH 7.0 were used in the preparation of a four-step density gradient, essentially as described by Mayilvaganan *et al*., 2001 [23]. The step gradient [15% (9 mL), 30% (9 mL), 50% (5 mL) and 60% (5 mL)] was prepared in ultra-clear centrifuge tubes and was overlaid with approximately 0.5 mL of partially purified phytoplasma preparation. Following centrifugation at 20,000 *g* for 20 min., the turbid zone visible in the tube containing infected leaf sample was collected and diluted with suspending medium. This was centrifuged again at 100,000 *g* for 120 min to remove Percoll. The pellet enriched with phytoplasma was collected in 1 mL of PBS, pH 7.0 and was dialyzed over night against PBS and finally clarified by centrifugation at 1,500 *g*. The phytoplasma enriched samples thus obtained were used for polyclonal antibody production. In this method all media were used at 4°C, as well as the centrifugation steps were carried at 4 ^o^C [23].

The protein content of the enriched phytoplasma preparation was determined using the micro well plate Bradford assay (Cat No. B6916, Sigma Aldrich, USA).

### Production of polyclonal antibodies

The protocol was approved by the Ethics Review Committee of the Faculty of Medicine, University of Colombo (Permit Number: EC-11-023). This study was carried out in strict accordance with the guidelines of this ethics review committee [24].

WCLWD associated phytoplasma specific polyclonal antiserum was raised in 2-3-month-old female New Zealand White rabbits (approximately 2.5 kg). All animals were of the same line and reared under uniform conditions at the Medical Research Institute, Sri Lanka.

Briefly, rabbits were immunized intramuscularly in the thigh muscle, with 1 mL of enriched phytoplasma preparation (2 mg/ mL of protein), and non-infected sample preparation for controls, emulsified with an equal volume of Freund’s complete adjuvant (primary dose). Subsequently, three such immunizations in Freund’s incomplete adjuvant were used as booster doses at fortnightly intervals [25]. Rabbits were bled from the marginal ear vein during the 7^th^ week after the initial immunization. Collected blood was allowed to clot at 37 ^o^C for 1 hour; then the clot was retracted from the wall of the tube and left at 4 ^o^C overnight. Serum was separated by centrifugation at 1000 *g* and stored at -20 ^o^C [26].

### Development of the Indirect ELISA

Indirect ELISA was developed essentially according to the method described by Sasikala et al. reported in 2005 with slight modifications [25]. Enriched phytoplasma preparation of WCLWD was used as the source of antigen, WCLWD specific antiserum as primary antibody, Goat anti-rabbit IgG conjugated with horseradish peroxidase (HRP) (Sigma Aldrich, Germany) as the enzyme conjugated secondary antibody and o-Phenylenediamine dihydrochloride (OPD) (Sigma Aldrich, Germany) as the chromogenic substrate. Reagent concentrations were optimized using standard checker board titrations.

Briefly, a 96 well microtiter plate (Immulon 2HB, Dynatech Laboratories, USA) was serially filled with 100 μl of test antigen (2mg/ml protein in IM PBS), 200 μl of blocking buffer (5% non-fat milk powder in 1M PBS), 100 μl of phytoplasma specific antiserum (1:1000) diluted in dilution buffer (0.5% Tween 20 in blocking buffer). Finally, 200 μl of the substrate (0.55mg OPD/ml citrate substrate buffer with 0.04% H_2_O_2_) was dispensed into each well. Between each step, the plate was incubated at 37°C for 1 hr and flick washed four times with washing buffer (0.5%Tween 20 in 1M PBS). The color reaction was allowed to proceed for 30 mins at room temperature (RT) and arrested with 1N H_2_SO_4_. The optical density was measured at 490 nm (OD_490_) in a Microplate Reader (Model M680, BioRad, USA).

Subsequently, indirect ELISA was established for 1:10 crude leaf extract as antigen. These extracts were prepared by grinding 1g of spear leaf sample in 10 mL of PBS (pH 7.4) containing 2% polyvinylpyrrolidone (PVP). The indirect ELISA steps detailed above wereperformed with the exception of using 1: 250 dilution of phytoplasma specific anti serum and washing buffer containing 1% of Tween 20.

The indirect ELISA based on crude coconut leaf extract as the source of antigen under optimized conditions was validated using phytoplasma positive and negative coconut leaf samples confirmed by *secA* based PCR. The cutoff value for the ELISA was set as the mean OD_490_ value of the *secA* PCR negative group plus two standard deviations (SD). Values of OD_490_ higher than the cut-off value were considered as test positives while those less than that were considered test negatives.

The non-parametric Mann-Whitney U test (IBM SPSS 21) was used for statistical comparison of ELISA results obtained from the groups of infected and non-infected coconut spear leaf samples.

### Validation of indirect ELISA

The minimum total number of crude leaf extracts to be screened was determined using the formula, *n* = {[4×*ds*×(1−*ds*)]/*e*^2^} where *ds* is the diagnosed sensitivity that is sought (95%) and *e* is the amount of error allowed in the estimate of diagnostic sensitivity (5%) [27]. The assay validation was performed considering *secA* based PCR as the ‘gold standard’, which refers to the method that gives unequivocal results [27]. Of 207 leaf samples obtained from different palms from the infected sites, those that scored positive in the *secA* based PCR (N = 197) were considered as disease positives whereas PCR negative leaf samples obtained from palms of disease non- prevalent areas (N = 192) were used as disease negative samples in the validation process.

The diagnostic accuracy of the established indirect ELISA was determined by receiver operating characteristic (ROC) curve analysis using IBM SPSS 21.0 to determine the accuracy [area under curve (AUC)] of the test to distinguish phytoplasma-positive from phytoplasma- negative palm samples, the diagnostic specificity, sensitivity and predictive values of positive (PPT) and negative (PNT) tests [27].

### ELISA cross reactivity

The cross reactivity of the developed WCLWD phytoplasma specific indirect ELISA with other phytoplasma strains was examined by replacing the coating antigen with crude extracts of leaves infected with SCWLD (N = 30), SCGSD (N = 25), AYLD (N = 25) and BGWLD (N = 35) confirmed by *secA* PCR. The degree of cross reactivity was expressed as the Cross-Reactivity Index (CRI) calculated as follows:

CRI=(nx/Nx)X100%

Where, n—number of leaf samples used as antigen, that reacted with WCLWD specific polyclonal anti serum (OD = ≥ 0.28)

N—Total number of leaf samples used to determine the cross reactivity

x—phytoplasma strain associated with the plant material (SCWLD, SCGSD, BGWLD, AYLD)

Testing the cross reactivity with antigens derived from other phytoplasma strains using the established ELISA was performed with Kruskal Wallis test followed by Mann-Whitney U test. P<0.05 was considered as significant.

#### Elucidation of ELISA cross reactivity by phylogenetic relationships, and by virtual restriction fragment length polymorphism (RFLP) analysis

Immunological cross reactivity reflects a close phylogenetic relationship between organisms, resulting in a high degree of homology in the 3-D structure of a specific protein, and thus potentially in immunological cross reactivity [28]. Therefore, the immunologic cross reactivity detected on the indirect ELISA of WCLWD associated phytoplasma with the other tested phytoplasma strains, was further investigated on their *secA* genes.

Partial *secA* sequences of phytoplasma strains associated with WCLWD, AYLD, SCWLD, SCGSD and BGWLD were compiled and aligned usingClustal W of Molecular Evolutionary Genetics Analysis 6.06 (MEGA 6.06) tool [29]. Next, the sequences were analyzed using the Maximum Likelihood method using Tamura 3- parameters model. The confidence level in phylogenetic analysis was determined using the bootstrap method, considering 1,000 bootstrap replications.

All sequences were exported to pDRAW32 developed by AcaClone Software (http://www.acaclone.com/) program for *in silico* digestion. Initially each DNA fragment was digested with 267 restriction enzymes available in pDRAW32 software of which 21distinct restriction enzymes (S1 Table) that gave maximum discrepancy between phytoplasma phylogenetic groups were selected [30].

Following enzyme digestion, a virtual agarose gel electrophoresis image was plotted. Settings of the virtual gel were accepted at 4% as the gel percentage, with minimum size of the bands at 10 bp and Invitrogen 25 bp DNA ladder as the DNA MW-marker [30]. The similarity coefficient (F) was calculated with the aid of the program written in Perl (Practical Extraction and Report Language) [31].

### Scanning electron microscopy (SEM)

Mid rib of the spear leaves of WCLWD infected and of non-infected coconut palms in which the phytoplasma presence / absence was verified by *secA* based PCR, were subjected to SEM. Small pieces (N = 5 each of disease positive and disease negative palms) of tissue were fixed first in 100% methanol for 10 min followed by 100% ethanol overnight [32]. Following critical point drying of samples, transverse sections were obtained by microtome SLEE Mainz (Cryostat MEV, Germany) at chamber temperature of -25 ^o^C. A finely cut transverse section (TS) (~10 μm) was placed on a carbon tape of an aluminium stub and subsequently sputter coated with gold. The tissue samples were observed using a scanning electron microscope (SU 6600 Hitachi, USA) in secondary electron mode at 10 keV accelerating voltage.

## Results

### PCR amplification of phytoplasma DNA

The nested PCR primed with RicesecAfor2/RicesecArev3, specific for phytoplasma groups 16SrXI and 16SrXIV produced the expected *secA* PCR amplicon of 420 bp. A total of 197 of 207 coconut palms (95%) with symptoms of WCLWD were confirmed as positive. None of the symptomless coconut samples screened positive, while all symptomatic sugarcane, Bermuda grass and areca nut leaf samples scored positive. All ten PCR amplicons that were sequenced (Accession No. KT719302.1—KT719306.1, KT345297.1—KT345300.1 and KM978909.1), indicated that the specific primers exclusively amplified the phytoplasma *secA* gene, but not bacterial DNA. Nucleotide BLAST of *secA* sequences confirmed the association of phytoplasma with WCLWD, closely related to phytoplasma strains of AYLD (KM978910.1) and BGWLD (KT369127.1- KT369134.1).

### Partial purification and enrichment of phytoplasma

Centrifugation of the Percoll-density gradient loaded with 0.5 mL of partially purified phytoplasma preparation resulted in a single, brown, opalescent turbid zone of 5–10 mm at the interphase of 15% and 30% step gradient of the discontinuous density gradient. Such a turbid zone was present in the tubes containing leaf samples infected with WCLWD, SCWLD, SCGSD, AYLD and BGWLD but clearly absent in those containing symptomless coconut leaf samples.

### Indirect ELISA

**Fig 1:**
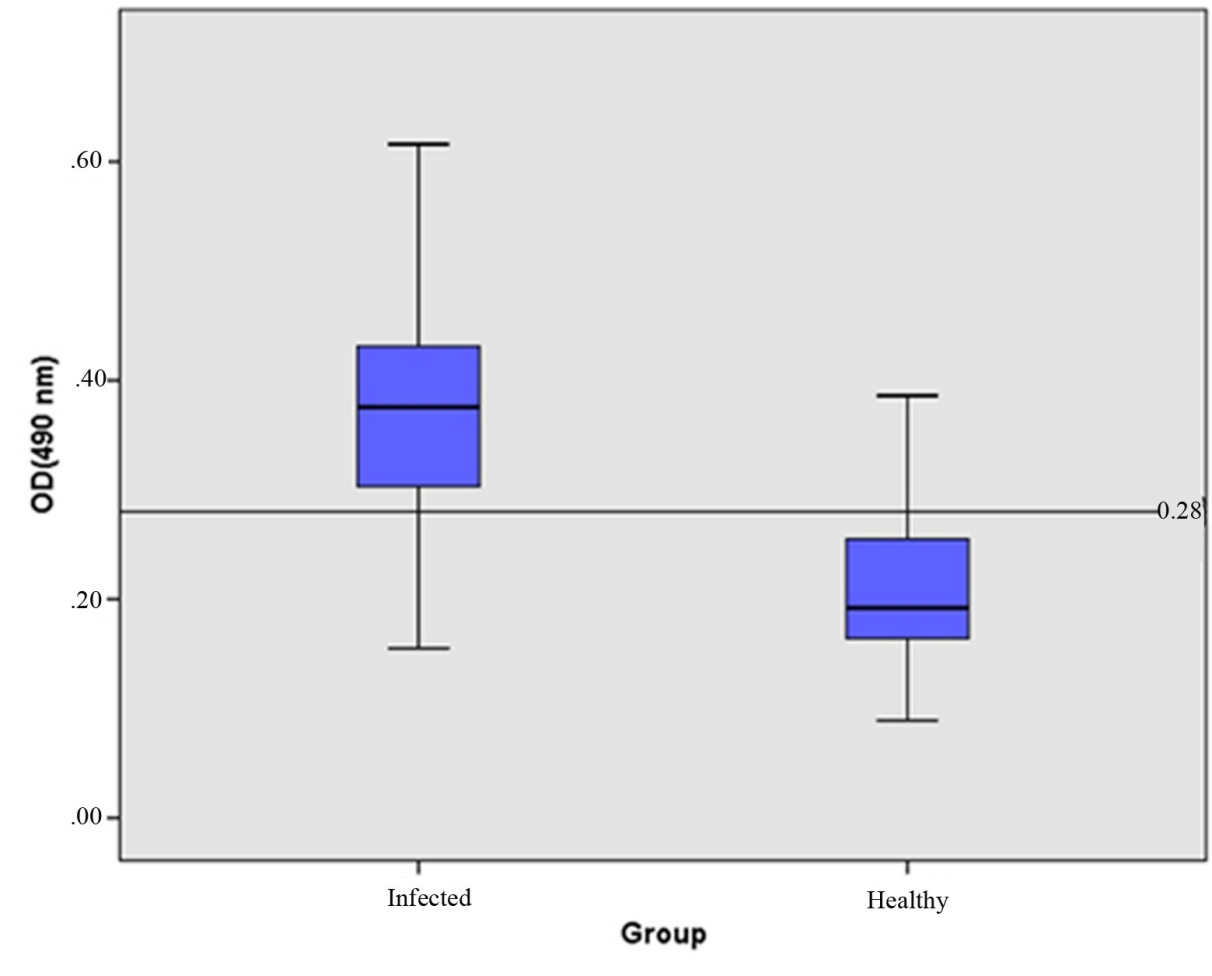
Indirect ELISA responses (OD_490_) of coconut samples symptomatic for WCLWD (N = 197) and of symptomless, apparently healthy coconut samples (N = 192). Standard box-plots with median, 25^th^ and 75^th^ percentiles and whiskers at maximum and minimum values are indicated. The horizontal line at y = 0.28 denotes the cut off value. *p < 0.05.

In accordance with The ELISA Guidebook, the significant minimum number of infected coconut palms to be tested in the established indirect ELISA was 76 [27], but prudently 197 symptomatic coconut palms were screened together with192 symptomless palms that had been tested by nested PCR of the *secA* gene. The cut off value of the ELISA was set at 0.28 (Fig 1).

Of the 197 PCR positive symptomatic coconut palms, 12 screened negative, while 35 PCR negative, symptomless palms scored positive by the established ELISA (Fig 1). Samples infected with WCLWD showed significantly higher OD_490_ values (0.155–0.644) than those of the non-infected palms (0.089–0.386) (p <0.05).

#### Validation of the indirect ELISA

**Fig 2:**
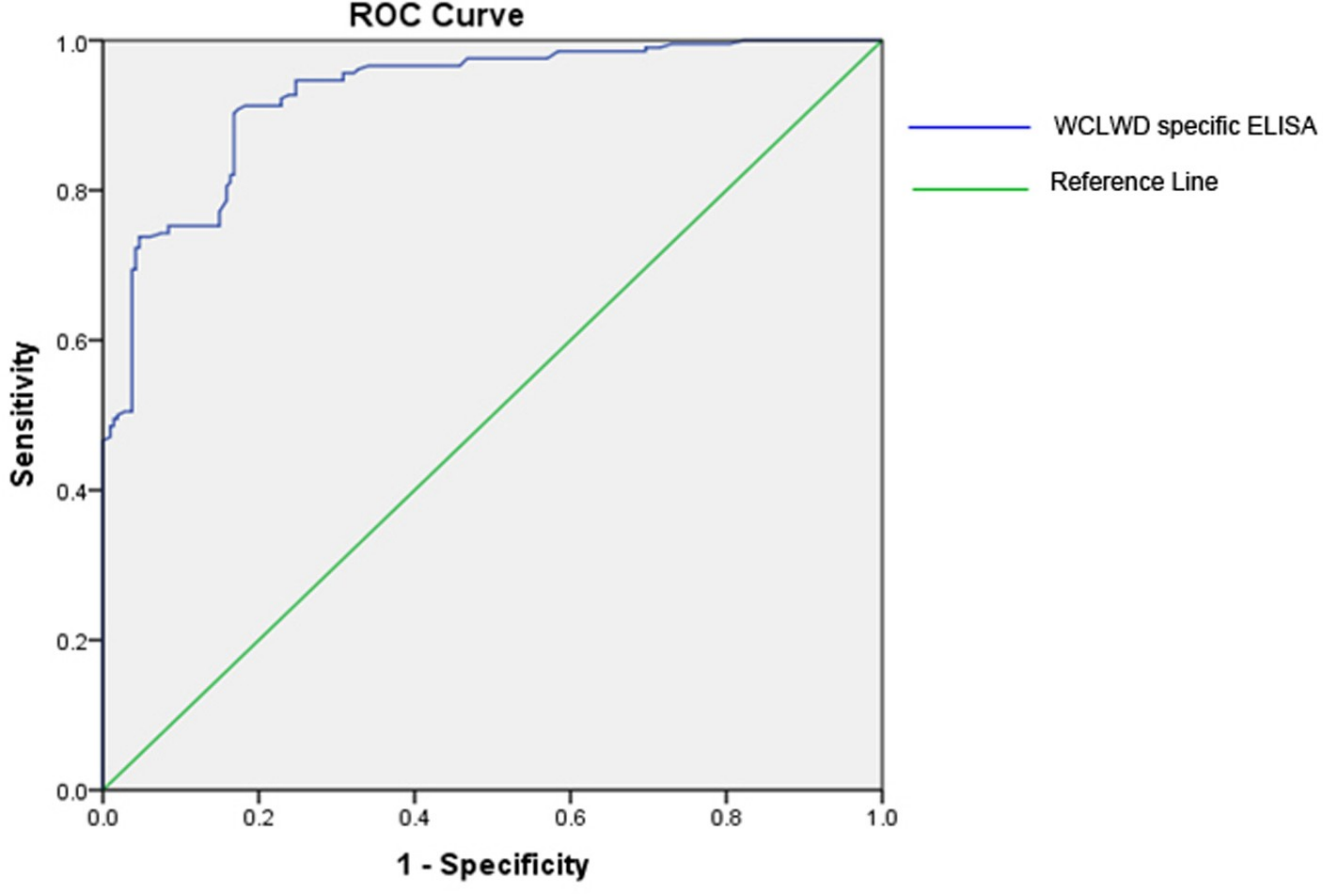
Receiver operating characteristic (ROC) curve analysis of the in house developed indirect ELISA to discriminate phytoplasma s*ecA* PCR positive coconut samples symptomatic for WCLWD from symptomless, apparently healthy coconut samples.

Receiver operating characteristic (ROC) curve analysis established a cut-off value of 0.279 with 92.7% sensitivity and79.5% specificity to distinguish WCLWD infected from non-infected coconut palms. Estimated area under the curve (0.928) indicated high accuracy (~93%) of the test in discriminating non-infected, symptomless palms from WCLWD infected palms (Fig 2). The respective values for false positivity and false negativity were 20.5% and 7.3%, while predictive value for positive and negative tests were 84.09% and 92.9%, respectively.

#### Cross reactivity of the indirect ELISA

**Fig 3:**
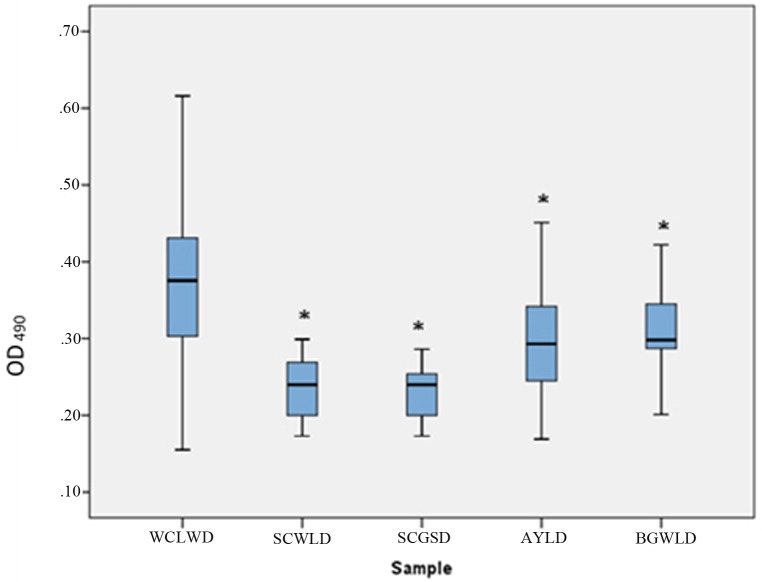
Indirect ELISA (OD_490_) responses of *sec A* PCR positive (i) WCLWD infected coconut samples (N = 197), (ii) sugarcane leaf samples infected with SCWLD(N = 30) and (iii) SCGSD (N = 25), (iv) leaves of Areca palms infected with AYLD (N = 25) and (v) Bermuda grass infected with BGWLD (N = 35). Standard box-plots with median, 25th and 75th percentiles and whiskers at maximum and minimum values are indicated. (*p < 0.05).

The established ELISA showed a considerable degree of cross reactivity with BGWLD (CRI = 69%) and with AYLD (70%) of phytoplasma group 16SrXIV. WCLWD specific polyclonal antiserum showed low cross reactivity with SCWLD (7%) and SCGSD (8%). However, there was a significant difference among the means of the ELISA absorbance values among the cross-reactive groups (Kruskal-Wallis test; p = 0.001). Pair wise comparison using Mann-Whitney U Test revealed significant differences between WCLWD / AYLD (p = 0.016), and WCLWD / BGWLD, SCWL, SCGS (p = 0.001) (Fig 3).

The ELISA cross reactivity was examined by *secA* gene based phylogenetic and RFLP analyses.

**Fig 4:**
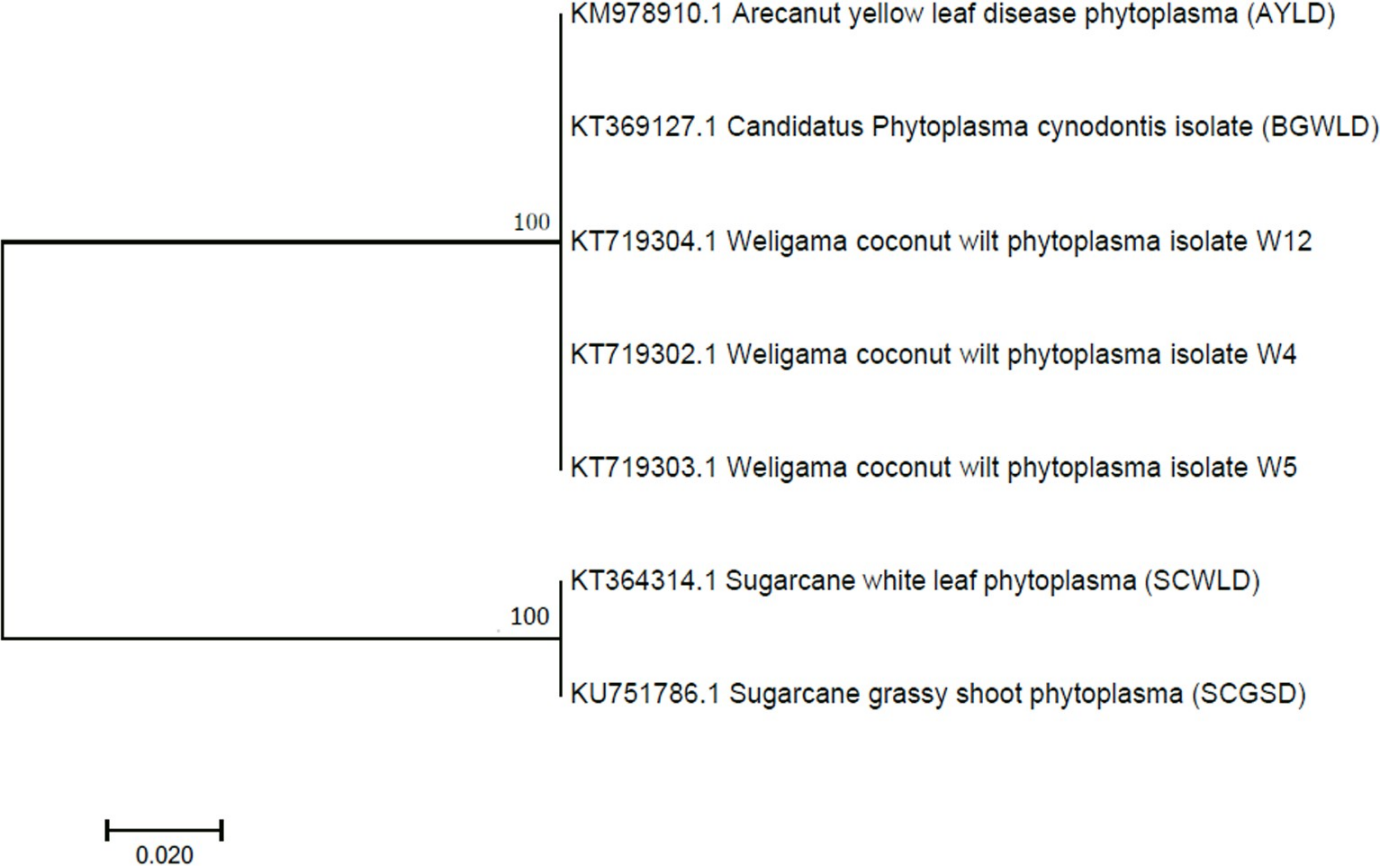
Maximum likelihood phylogram based on partial *secA* sequences of WCLWD, AYLD, SCWLD, SCGSD and BGWLD associated phytoplasma strains. A bootstrap test of 1,000 replication was performed to examine the reliability of the phylogeny. Numbers at nodes indicate percentage bootstrap support probability values. The GenBank accession number is preceded by the phytoplasma name.

The maximum likelihood phylogenetic tree (Fig 4) based on *secA* sequences of phytoplasma strains associated with WCLWD, AYLD, SCWLD, SCGSD and BGWLD revealed that WCLWD and AYLD associated phytoplasma strains that are identical to each other [33] clustered with BGWLD phytoplasma of the 16SrXIV group ‘*Candidatus* Phytoplasma cynodontis’ (boostrap value; 99), showing nonconformity with the exisitng classification based on the 16S rRNA gene [7], while the used sugarcane phytoplasmas showed a clear seperation from the above cluster.

The initial digestion of WCLWD, AYLD, BGWLD, SCWLD and SCGSD phytoplasma *secA* sequences with the complete panel of 281 restriction enzymes available at the pDRAW32 virtual RFLP analysis tool, revealed that only 21 of these enzymes had the potential to differentiate phytoplasma strains of group 16SrXIV from those of the 16SrXI group.

**Fig 5:**
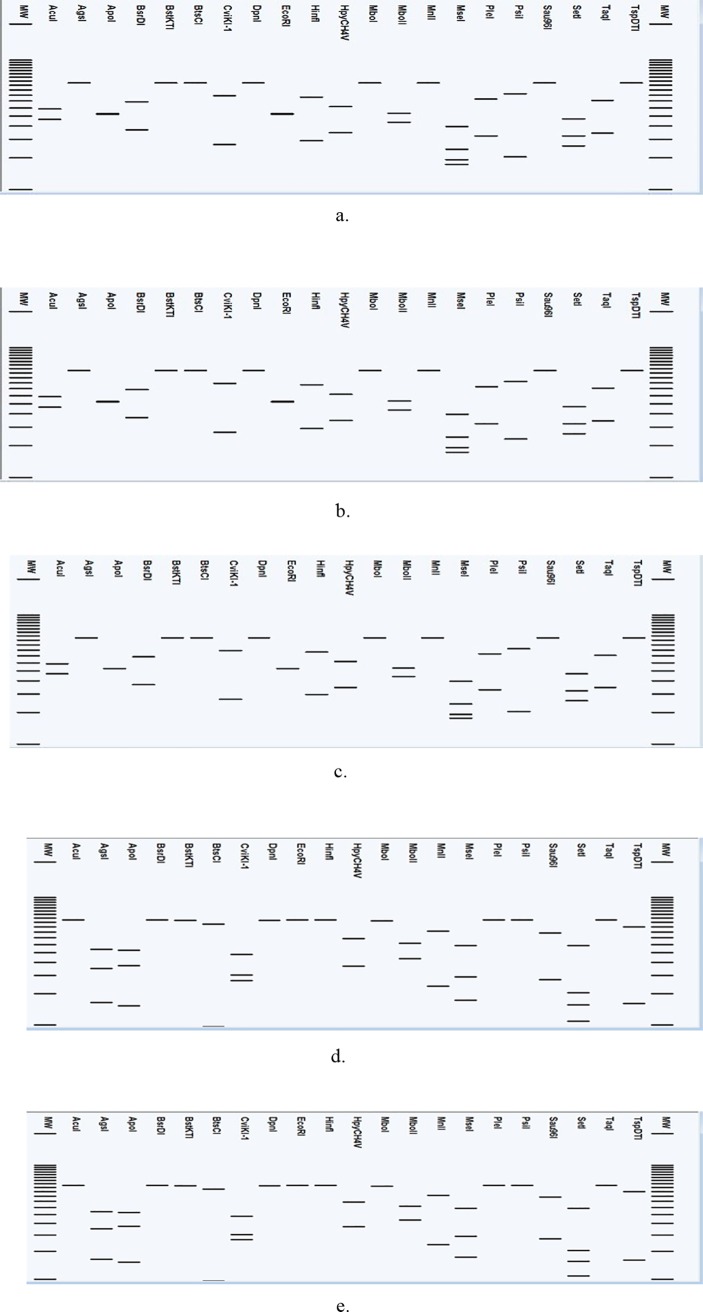
**Virtual RFLP gel patterns derived from *in silico* digestion of *secA* (264 bp) of (a) WCLWD phytoplasma and other phytoplasma strains prevalent in Sri Lanka associated with (b) AYLD, (c) BGWLD, (d) SCWLD and (e) SCGSD.** Sequences were digested using 21 restriction endonuclease enzymes; *AcuI*, *AgsI*, *ApoI*, *BsrDI*, *BstKTI*, *BtsCI*, *CviKI-1*, *DpnI*, *EcoRI*, *HinfI*, *HpyCH4V*, *MboI*, *MboII*, *MnlI*, *MseI*, *PleI*, *PsiI*, *Sau96I*, *SetI*, *TaqI*, *TspDTI*. MW—25 bp step ladder (Promega).

The virtual gel diagrams generated by *in silico* RFLP analysis of the *secA* indicated that WCLWD and the AYLD phytoplasma, were closely related to the BGWLD phytoplasma (Fig 5). The similarity coefficient (F) calculated for the above five phytoplasma strains confirmed that WCLWD and AYLD phytoplasmas were indeed closely related to BGWLD phytoplasma (F = 1.0). Conversely, similarity coefficient factor calculated between WCLWD and sugarcane phytoplasma strains (SCWLD and SCGSD) indicated a significant dissimilarity (F = 0.56).

Thus, the outcome of both the phylogenetic and the RFLP analyses of the five phytoplasma strains tested, clarified the immunological cross reactivity they showed in the indirect ELISA.

### Scanning electron microscopy (SEM)

**Fig 6:**
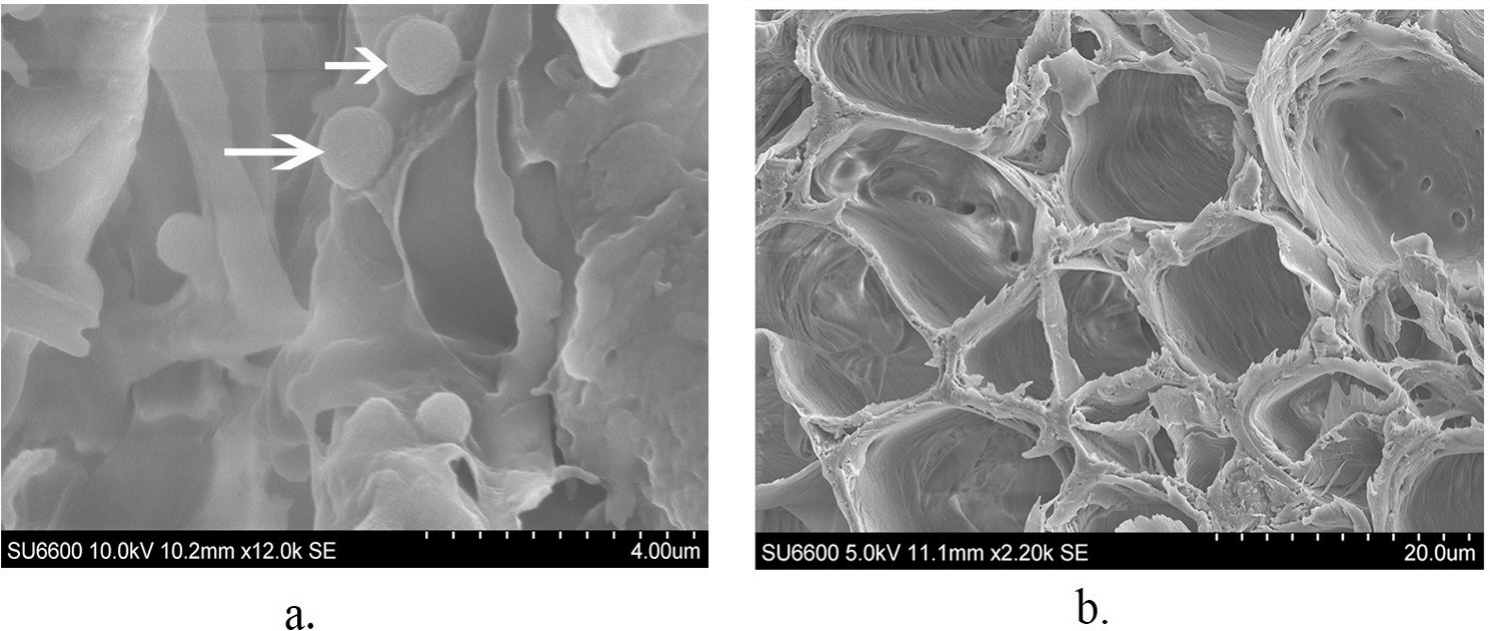
**Scanning electron micrographs of transverse sections of phloem tissue (a) infected with WCLWD associated phytoplasma (b) of non-infected, coconut pinnae mid rib samples devoid of such phytoplasma bodies**. Spherical, pleomorphic (200–1000 nm) phytoplasma bodies are visible, attached to the inner surface of the cellular membrane of the infected sieve elements (indicated by arrows).

Observations of cross sections of mid rib of leaflets (ekel) of infected spear leaves by SEM revealed the presence of spherical, pleomorphic phytoplasma-like bodies ranging in diameter from 200 to 1000 nm (Fig 6A). These appeared as separate or clustered particles in diseased plants that were adherent onto the inner surface of the sieve tubes. The uneven distribution of phytoplasmas-like bodies in sieve tubes was noted. These bodies were absent in the cross sections of ekel of asymptomatic coconut samples (Fig 6B).

## Discussion

Phytoplasma are associated with disease symptoms in coconut palms in different geographical regions in the world and are increasingly being associated with other palms as well [34]. Most phytoplasma associated diseases cause serious threats to the sustainability of coconut cultivation, worldwide.

Phytoplasma associated WCLWD has emerged as the most perilous threat to the coconut cultivation in the southern province of Sri Lanka [4]. A previous study recorded *Goniagnathus punctifer* (T.), *Recilia dorsalis* (Motschulsky), *Kolla ceylonica* (Melichar), *Idioscopus clypealis* (Lethierry), *Proutista moesta* (Westwood), *Proutista* sp., *Nisia nervosa* (Motschulsky) and an unknown Cixiid and *Stephanitis typica* (Distant) as some of the putative insect vectors involved in transmission of WCLWD [35]. Due to the significance of the disease, rapid and accurate diagnosis of phytoplasma presence, strain differentiation, identification of alternative hosts is essential to support disease surveillance, and timing of interventions to mitigate the disease spread.

Though phytoplasma enrichment was achieved it was not possible to produce a completely pure phytoplasma preparation. Thus far, isolation of pure phytoplasma preparations free of plant host tissue has not been achieved by any means, and in order to circumvent this problem, Shahryari et al (2013) propose the use of recombinant phytoplasma proteins to produce anti-phytoplasma antibodies [15]. The cut off value of the indirect ELISA was based on the OD values obtained for the asymptomatic, apparently healthy palm material as the antigen source, which logically should have contained host tissue components. Thus, it was assumed that the “background” signal by the antibodies against host plant tissue was negated by the cut off value set for the indirect ELISA.

The discriminative potential or the accuracy of a diagnostic method is quantified by measuring its diagnostic sensitivity, specificity, and predictive positive and negative values of the assay [36]. The diagnostic accuracy of the assay was calculated using Receiver Operating Characteristic Curve (ROC) analysis which is a common approach for displaying the discriminatory power of a diagnostic test [37]. The area under the curve (AUC), is a quantitative and descriptive expression of how close the ROC curve is to the perfect one (AUC = 1.0). AUC for the developed ELISA was 0.928, indicative of high accuracy (~93%) to discriminate between non-infected and infected samples.

The established ELISA showed significant performance in terms of diagnostic sensitivity (92.7%). However, the high false positivity (20.5%) of this assay hinders the immediate introduction of this assay as a field level diagnostic method. Yet development of a diagnostic Indirect ELISA based on specific monoclonal antibodies to conserved antigenic epitopes of the WCLWD associated phytoplasma would be promising.

A close phylogenetic relationship between WCLWD and SCWLD associated phytoplasmas was evident based on the 16SrRNA gene [4]. Conversely, we observed a low CRI between these two phytoplasma strains on the indirect ELISA, which was reiterated by *secA* based phylogenetic and RFLP analyses. Furthermore, the high CRI among WCLWD, AYLD and BGWLD associated phytoplasma strains in the indirect ELISA was supported by the *in silico* RFLP banding pattern and the similarity coefficient values based on the *secA* gene. Conversely, based on the 16S rRNA gene, WCLWD and BGWLD phytoplasma strains are classified under 16SrXIand 16SrXIV groups, respectively. Therefore, the need to use other, relatively less conserved protein coding genetic markers such as ribosomal protein (rp) genes, *secY*, *tu* collectively with *secA* to determine the phylogenetic relationship of phytoplasma strains is imperative [38].

This study for the first time shows the presence of possible phytoplasma like bodies in WCLWD infected tissues by scanning electron microscopy.

## Conclusion

A validated polyclonal antibody based indirect ELISA, which is a potential, rapid immunodiagnostic in the pipeline for WCLWD associated phytoplasma is reported. Yet the routine use of this indirect ELISA was hindered due to its relatively high false positivity. Nevertheless, this will pave the way to establish a serviceable indirect ELISA for the detection of the WCLWD associated phytoplasma, based on specific monoclonal antibodies produced against this phytoplasma strain.

## Supporting information

S1 Table Predicted sizes for *secA* gene fragments following digestion with *AcuI*, *AgsI*, *ApoI*, *BsrDI*, *BstKTI*, *BtsCI*, *CviKI-1*, *DpnI*, *EcoRI*, *HinfI*, *HpyCH4V*, *MboI*, *MboII*, *MnlI*, *MseI*, *PleI*, *PsiI*, *Sau96I*, *SetI*, *TaqI*, *TspDTI*. (DOCX)
