# Supplementary material for: Immunological detection of the Weligama coconut leaf wilt disease associated phytoplasma: Development and validation of a polyclonal antibody based indirect ELISA
Source: PLoS One. 2019 Apr 9;14(4):e0214983. doi: 10.1371/journal.pone.0214983 (PMC6456191; doi:10.1371/journal.pone.0214983)
Supplement: S1 Table — (DOCX) [file pone.0214983.s001.docx]

| **Phytoplasma strain** | **Predicted**  **Fragment size (bp)** | | | | |
| --- | --- | --- | --- | --- | --- |
|  | **WCLWD** | **AYLD** | **BGWLD** | **SCWLD** | **SCGSD** |
| ***Uncut*** | 263 | 263 | 264 | 263 | 263 |
| ***AcuI*** | 116, 147 | 116, 147 | 117, 147 | 263 | 263 |
| ***AgsI*** | 263 | 263 | 264 | 88, 41, 134 | 88, 41, 134 |
| ***ApoI*** | 131, 132 | 131, 132 | 132, 132 | 38, 93, 132 | 38, 93, 132 |
| ***BsrDI*** | 171, 92 | 171, 92 | 172, 92 | 263 | 263 |
| ***BstKTI*** | 263 | 263 | 264 | 261, 2 | 261, 2 |
| ***BtsCI*** | 263 | 263 | 264 | 24, 239 | 24, 239 |
| ***CviKI-1*** | 196, 67 | 196, 67 | 197, 67 | 76, 120, 67 | 76, 120, 67 |
| ***DpnI*** | 263 | 263 | 264 | 260, 3 | 260, 3 |
| ***EcoRI*** | 131, 132 | 131, 132 | 132, 132 | 263 | 263 |
| ***HinfI*** | 73, 190 | 73, 190 | 74, 190 | 263 | 263 |
| ***HpyCH4V*** | 22, 154, 87 | 22, 154, 87 | 22, 155, 87 | 171, 92 | 171, 92 |
| ***MboI*** | 263 | 263 | 264 | 258, 5 | 258, 5 |
| ***MboII*** | 109, 21, 133 | 109, 21, 133 | 110, 21, 133 | 109, 154 | 109, 154 |
| ***MnlI*** | 263 | 263 | 264 | 59, 204 | 59, 204 |
| ***MseI*** | 43, 48, 99, 60, 13 | 43, 48, 99, 60, 13 | 44, 48, 99, 60, 13 | 43, 147, 73 | 43, 147, 73 |
| ***PleI*** | 81, 182 | 81, 182 | 82, 182 | 263 | 263 |
| ***PsiI*** | 205, 51, 7 | 205, 51, 7 | 206, 51, 7 | 263 | 263 |
| ***Sau96I*** | 263 | 263 | 264 | 195, 68 | 195, 68 |
| ***SetI*** | 117, 81, 65 | 117, 81, 65 | 118, 81, 65 | 51, 27, 39, 146 | 51, 27, 39, 146 |
| ***TaqI*** | 86, 177 | 86, 177 | 87, 177 | 263 | 263 |
| ***TspDTI*** | 263 | 263 | 264 | 223, 40 | 223, 40 |

**S1 Table. Predicted sizes for secA gene fragments following digestion with *AcuI, AgsI, ApoI, BsrDI, BstKTI, BtsCI, CviKI-1, DpnI, EcoRI, HinfI, HpyCH4V, MboI, MboII, MnlI, MseI, PleI, PsiI, Sau96I, SetI, TaqI, TspDTI***
